# Supplementary material for: Identification and Evolutionary Relationship of Corynebacterium striatum Clinical Isolates
Source: Pathogens. 2022 Sep 5;11(9):1012. doi: 10.3390/pathogens11091012 (PMC9501166; doi:10.3390/pathogens11091012)
Supplement: Supplementary file 1 [file pathogens-11-01012-s001.zip › pathogens-1880178-supplementary.pdf]

Table S1. Clinical information of Specimens.

Number	Hosptial Unit	Sample time (month/day/year)	Specimen type	Clinical diagnosis	Number	Hosptial Unit	Sample time (month/day/year)	Specimen type	Clinical diagnosis
Cs-1	Critical Care Medicine	3/29/2019	sputum	gastrointestinal hemorrhage	Cs-15	Orthopedics	6/12/2019	sputum	cerebral infarction
Cs-7	Critical Care Medicine	1/5/2019	sputum	yet to be investigated	Cs-18	Orthopedics	6/6/2019	excretion	chronic infection of right hip
Cs-8	Critical Care Medicine	4/15/2019	sputum	right basal ganglia	Cs-19	Orthopedics	7/8/2019	excretion	skin removal area of right thigh
Cs-36	Critical Care Medicine	5/10/2020	excretion	cerebral infarction	Cs-24	Orthopedics	7/10/2019	excretion	soft tissue ulceration of left foot
Cs-25	Critical Care Medicine	8/14/2019	sputum	massive hemorrhage of gastrointestinal tract	Cs-27	Orthopedics	8/27/2019	excretion	chronic infection of right hip
Cs-4	Neurology	12/13/2018	sputum	large-area cerebral infarction	Cs-28	Orthopedics	8/23/2019	excretion	sacrococcygeal pressure sores
Cs-5	Neurology	12/18/2018	excretion	Left temporal lobe subdural	Cs-30	Orthopedics	11/27/2019	excretion	sacrococcygeal pressure sores
Cs-9	Neurology	1/15/2019	blood	co poisoning	Cs-32	Orthopedics	11/13/2019	excretion	skin and soft tissue injury of right calf
Cs-10	Neurology	12/13/2018	sputum	the cause of the disturbance of consciousness is unknown	Cs-34	Orthopedics	12/20/2019	excretion	open fracture of right foot
Cs-12	Neurology	12/13/2018	sputum	the cause of the disturbance of consciousness is unknown	Cs-35	Orthopedics	1/16/2020	excretion	bacteremia
Cs-17	Neurology	7/24/2019	sputum	the cause of the disturbance of consciousness is unknown	Cs-37	Orthopedics	5/10/2020	excretion	arterial embolization in the left lower limb
Cs-23	Neurology	7/11/2019	sputum	the cause of the disturbance of consciousness is unknown	Cs-38	Orthopedics	5/23/2020	excretion	craniocerebral injury
Cs-39	Neurology	5/23/2020	sputum	dizziness paralysis of half right limb	Cs-42	Orthopedics	6/15/2020	excretion	pilonidal sinus cysts were hidden in the sacrococcygeal region
Cs-3	Nephrology	2/19/2019	urine	chronic kidney diseases	Cs-45	Orthopedics	7/8/2020	excretion	sacrococcygeal pressure ulcer
Cs-6	Nephrology	12/18/2018	blood	yet to be investigated	Cs-46	Orthopedics	7/21/2020	excretion	sacrococcygeal pressure ulcer
Cs-21	Nephrology	8/3/2019	excretion	type 2 diabetes	Cs-47	Orthopedics	7/22/2020	excretion	systemic lupus erythematosus
Cs-22	Nephrology	8/1/2019	urine	nephrotic syndrome	Cs-49	Orthopedics	7/28/2020	excretion	right diabetic foot
Cs-40	Nephrology	6/3/2020	blood	chronic kidney disease stage 5	Cs-50	Orthopedics	7/30/2020	excretion	pressure sore

Cs-41	Nephrology	6/4/2020	blood	chronic kidney disease stage 5	Cs-51	Orthopedics	7/30/2020	excretion	sacrococcygeal pressure ulcer
Cs-2	Emergency ICU	4/25/2019	excretion	aortic dissection	Cs-52	Orthopedics	8/7/2020	excretion	sacrococcygeal pressure ulcer
Cs-13	Obstetrics	7/30/2019	excretion	abdominal wall incision after cesarean section	Cs-53	Orthopedics	8/11/2020	excretion	left interfemoral intercalation
Cs-14	Endocrinology	6/13/2019	excretion	type 2 diabetes	Cs-54	Orthopedics	8/14/2020	excretion	pressure sores in the pressure zone
Cs-16	Traditional Chinese Medicine	6/14/2019	urine	stroke	Cs-11	Neurosurgery	1/16/2019	sputum	right thalamic hemorrhage
Cs-26	Head and Neck Oncology	8/22/2019	excretion	squamous cell carcinoma of the scalp	Cs-20	Neurosurgery	6/21/2019	sputum	yet to be investigated
Cs-29	Gynecology	8/29/2019	excretion	serous carcinoma of the right ovary	Cs-43	Neurosurgery	6/21/2020	sputum	acute craniocerebral injury
Cs-31	County Hospital (Baokang)	11/10/2019	excretion	yet to be investigated	Cs-33	Respiratory Medicine	12/8/2019	excretion	pneumonia
Cs-48	Otorhinolaryngology head and neck surgery	7/25/2020	excretion	new laryngeal organisms to be investigated	Cs-44	Respiratory Medicine	6/27/2020	sputum	community-acquired pneumonia

Note: the sample sources of the same department are marked with the same colour shade. The strain's sample information containing the *integrase* gene is underlined.

Table S2. The minimum inhibitory concentrations of antimicrobial agents against *C. striatum* isolates.

Number	Penicillin	Ceftriaxone	Vancomycin	Clindamycin	Eeythromycin	Ciprofloxacin	Linezolid	Tetracycline	Meropenem
Cs-1	=2/I	=4/R	≤1/S	=1/I	≥16/R	≥4/R	≤2/S	=32/R	≤0.25/S
Cs-2	≥32/R	≥8/R	≤1/S	≥8/R	≥16/R	≥4/R	≤2/S	=32/R	≥2/R
Cs-3	=8/R	≥8/R	≤1/S	≥8/R	≥16/R	≥4/R	≤2/S	=32/R	≥2/R
Cs-4	≥32/R	≥8/R	≤1/S	≥8/R	=8/R	≥4/R	≤2/S	=32/R	≥1/R
Cs-5	≥32/R	≥8/R	≤1/S	≥8/R	≥16/R	≥4/R	≤2/S	=32/R	≥2/R
Cs-6	≥32/R	≥8/R	≤1/S	≥8/R	≥16/R	≥4/R	≤2/S	=32/R	≥2/R
Cs-7	≥32/R	≥8/R	≤1/S	≥8/R	≥16/R	≥4/R	≤2/S	=32/R	≥2/R
Cs-8	=2/I	=4/R	≤1/S	=1/I	≥16/R	≥4/R	≤2/S	=32/R	≤0.25/S
Cs-9	=2/I	=4/R	≤1/S	=1/I	≥16/R	≥4/R	≤2/S	=32/R	≤0.25/S
Cs-10	=2/I	=4/R	≤1/S	=1/I	≥16/R	≥4/R	≤2/S	=32/R	≤0.25/S
Cs-11	≥32/R	≥8/R	≤1/S	≥8/R	≥16/R	≥4/R	≤2/S	=32/R	≥1/R
Cs-12	≥32/R	≥8/R	≤1/S	≥8/R	≥16/R	≥4/R	≤2/S	=32/R	≥2/R
Cs-13	=2/I	=2/I	≤1/S	≥8/R	≥16/R	=2/I	≤2/S	≤1/S	≥2/R
Cs-14	≥32/R	≥8/R	≤1/S	≥8/R	≥16/R	≥4/R	≤2/S	=32/R	=1/R
Cs-15	≥32/R	≥8/R	≤1/S	≥8/R	≥16/R	≥4/R	≤2/S	=32/R	≥2/R
Cs-16	=2/I	=4/R	≤1/S	=0.5/S	=1/I	≥4/R	≤2/S	=32/R	≤0.25/S
Cs-17	≥32/R	≥8/R	≤1/S	≥8/R	≥16/R	≥4/R	≤2/S	=32/R	≥2/R
Cs-18	≥32/R	≥8/R	≤1/S	≥8/R	≥16/R	≥4/R	≤2/S	=32/R	≥2/R
Cs-19	=0.12/S	=2/I	≤1/S	≥8/R	≥16/R	=2/I	≤2/S	≤1/S	≥2/R
Cs-20	=2/I	≥8/R	≤1/S	=1/I	≥16/R	≥4/R	≤2/S	=32/R	≤0.25/S
Cs-21	=2/I	≥8/R	≤1/S	=1/I	≥16/R	≥4/R	≤2/S	=32/R	≤0.25/S
Cs-22	≥32/R	≥8/R	≤1/S	≥8/R	≥16/R	≥4/R	≤2/S	=32/R	≥2/R
Cs-23	≥32/R	≥8/R	≤1/S	≥8/R	≥16/R	≥4/R	≤2/S	=32/R	≥2/R
Cs-24	=2/I	≥8/R	≤1/S	=1/I	≥16/R	≥4/R	≤2/S	=32/R	≤0.25/S
Cs-25	≥32/R	≥8/R	≤1/S	≥8/R	≥16/R	≥4/R	≤2/S	=32/R	≥2/R
Cs-26	=8/R	≥8/R	≤1/S	≥8/R	≥16/R	≥4/R	≤2/S	=8/I	=0.5/I
Cs-27	=8/R	≥8/R	≤1/S	≥8/R	≥16/R	≥4/R	≤2/S	=32/R	≥2/R
Cs-28	=2/I	≥8/R	≤1/S	≥8/R	≤0.25/S	≥4/R	≤2/S	=32/R	≤0.25/S
Cs-29	=0.12/S	=2/I	≤1/S	≥8/R	≥16/R	≥4/R	≤2/S	≤1/S	≤0.25/S
Cs-30	=4/R	≥8/R	≤1/S	=4/R	≤0.25/S	≥4/R	≤2/S	=32/R	≤0.25/S
Cs-31	≥32/R	≥8/R	≤1/S	=4/R	=0.5/S	≥4/R	≤2/S	=32/R	≥2/R
Cs-32	=4/R	≥8/R	≤1/S	=4/R	≤0.25/S	≥4/R	≤2/S	=32/R	≤0.25/S
Cs-33	=8/R	≥8/R	≤1/S	≥8/R	≥16/R	≥4/R	≤2/S	=32/R	≥2/R
Cs-34	≥32/R	≥8/R	≤1/S	=4/R	=4/R	≥4/R	≤2/S	=32/R	≥2/R
Cs-35	≥32/R	≥8/R	≤1/S	=4/R	=4/R	≥4/R	≤2/S	=32/R	≥2/R
Cs-36	≥32/R	≥8/R	≤1/S	≥8/R	=8/R	≥4/R	≤2/S	=8/I	≥2/R
Cs-37	=16/R	≥8/R	≤1/S	≥8/R	≥16/R	≥4/R	≤2/S	=32/R	≥2/R
Cs-38	≥32/R	≥8/R	≤1/S	≥8/R	≥16/R	≥4/R	≤2/S	=32/R	≥2/R
Cs-39	≥32/R	≥8/R	≤1/S	≥8/R	≥16/R	≥4/R	≤2/S	=32/R	≥2/R
Cs-40	≥32/R	≥8/R	≤1/S	≥8/R	≥16/R	≥4/R	≤2/S	=32/R	≥2/R
Cs-41	≥32/R	≥8/R	≤1/S	≥8/R	=4/R	≥4/R	≤2/S	=32/R	≥2/R
Cs-42	=16/R	≥8/R	≤1/S	≥8/R	=4/R	≥4/R	≤2/S	=32/R	≥2/R

Cs-43	≥32/R	≥8/R	≤1/S	≥8/R	≥16/R	≥4/R	≤2/S	=32/R	≥2/R
Cs-44	≥32/R	≥8/R	≤1/S	≥8/R	=4/R	≥4/R	≤2/S	=32/R	≥2/R
Cs-45	=16/R	≥8/R	≤1/S	≥8/R	≥16/R	≥4/R	≤2/S	=32/R	≥2/R
Cs-46	=16/R	≥8/R	≤1/S	≥8/R	≥16/R	≥4/R	≤2/S	=32/R	≥2/R
Cs-47	=16/R	≥8/R	≤1/S	≥8/R	≥16/R	≥4/R	≤2/S	=32/R	≥2/R
Cs-48	=8/R	≥8/R	≤1/S	≥8/R	≥16/R	≥4/R	≤2/S	=32/R	≥2/R
Cs-49	=16/R	≥8/R	≤1/S	≥8/R	≥16/R	≥4/R	≤2/S	=32/R	≥2/R
Cs-50	=16/R	≥8/R	≤1/S	≥8/R	≥16/R	≥4/R	≤2/S	=32/R	≥2/R
Cs-51	=16/R	≥8/R	≤1/S	≥8/R	≥16/R	≥4/R	≤2/S	=32/R	≥2/R
Cs-52	=16/R	≥8/R	≤1/S	≥8/R	≥16/R	≥4/R	≤2/S	=32/R	≥2/R
Cs-53	=16/R	≥8/R	≤1/S	≥8/R	≥16/R	≥4/R	≤2/S	=32/R	≥2/R
Cs-54	≥32/R	≥8/R	≤1/S	≥8/R	≥16/R	≥4/R	≤2/S	=32/R	≥2/R

Note: MIC/(μg/mL); R, resistant; S, susceptible; I, intermediate.

Table S3. PCR primers used in the study.

Gene	Name	Sequence (5' - 3')	Size of products（bp）
16s rRNA[1]	16SF27	AGAGTTTGATCMTGGCTCAG	~ 1500
	16SR1492	TACGGYTACCTTGTTACGACTT	
integrase	Int-1151-F	ATGGCAAGGAAGAAACTACCG	1151
	Int-1151-R	AGTTCTAGGATTGCCGTGTGAT	

[1]Suh JW, Ju Y, Lee CK, Sohn JW, Kim MJ, Yoon YK: Molecular epidemiology and clinical significance of *Corynebacterium striatum* isolated from clinical specimens. Infect Drug Resist 2019, 12:161-171.

```
Int_Cs216      1 MARKKLPIGELGTIKFTQVGPRKWRARGYVRTYSGTRVQVQGTGRTKGIA    50
                 ||||||||||||||||||||||||||||||||||||||||||||||||||
Int_Cs         1 MARKKLPIGELGTIKFTQVGPRKWRARGYVRTYSGTRVQVQGTGRTKGIA    50

Int_Cs216     51 EQTLRTNANMRVYENAGALLDSNSTLNELLRQTLDAMRAGTIGKKLRVQS   100
                 |||||||||||||||||||||||||||||||||||||||||:||||||||
Int_Cs        51 EQTLRTNANMRVYENAGALLDSNSTLNELLRQTLDAMRAGTVGKKLRVQS   100

Int_Cs216    101 VNTYERQLSLFKGERGDQAIGNLPLYECSKNVLTHWLMKLSERTPANAKL   150
                 |||||||||||||||||||||||||||||||||||||||:|||||.||||
Int_Cs       101 VNTYERQLSLFKGERGDQAIGNLPLYECSKNVLTHWLMKVSERTPGNAKL   150

Int_Cs216    151 AKVLLSRAYDLTAMHGLEIWTSNPTYGVKLHSKDKENDEPVALSLADIQT   200
                 ||||||||||||||||||||||||||||||||||||||||||||||||||
Int_Cs       151 AKVLLSRAYDLTAMHGLEIWTSNPTYGVKLHSKDKENDEPVALSLADIQT   200

Int_Cs216    201 IWQNVQAWQTDYKRTDLVGVVGACMATGFRINEVLALQWADIDLSTSPAT   250
                 ||||||||||||||||||||||||||||||||||||||||||||||||||
Int_Cs       201 IWQNVQAWQTDYKRTDLVGVVGACMATGFRINEVLALQWADIDLSTSPAT   250

Int_Cs216    251 ITNTGTLVRQDGKLIRQPKTKTKNGFRVVKIPEWFADMLRARVARADSPL   300
                 ||||||||||||||||||||||||||||||||||||||||.|:|||||||
Int_Cs       251 ITNTGTLVRQDGKLIRQPKTKTKNGFRVVKIPEWFADMLRVRIARADSPL   300

Int_Cs216    301 VFPNDRGGFMDAVNIRTRFREARGPMFEHVKFKSFRSSVATTIANTTSVE   350
                 ||||||||||||||||||||||||||||||||||||||||||||||||||
Int_Cs       301 VFPNDRGGFMDAVNIRTRFREARGPMFEHVKFKSFRSSVATTIANTTSVE   350

Int_Cs216    351 EAQKQLGHSSPNITQRYYVQRAADAGDHTAILELFAPANVMIK*    394
                 |||||||||||||||||||||||||||||||||||||||||||
Int_Cs       351 EAQKQLGHSSPNITQRYYVQRAADAGDHTAILELFAPANVMIK*    394
```

Figure S1. Amino acid sequence of integrase alignments between MDR-*C. striatum* and *C. striatum* 216 (WP_100619009.1).
